# Peripheral kynurenine-3-monooxygenase deficiency as a potential risk factor for metabolic syndrome in schizophrenia patients

**DOI:** 10.15761/ICM.1000105

**Published:** 2017-05-10

**Authors:** Gregory Oxenkrug, Marieke van der Hart, Julien Roeser, Paul Summergrad

**Affiliations:** 1 Department of Psychiatry, Tufts University School of Medicine, USA; 2 Brains On-Line, S. San Francisco, USA

**Keywords:** Kynurenine-3-monooxygenase, Kynurenine, Kynurenic acid, Anthranilic Acid, Tryptophan, Schizophrenia, Obesity, Diabetes, Metabolic syndrome, Aryl Hydrocarbon receptor

## Abstract

Increased predisposition of schizophrenia patients (SP) to development of obesity and insulin resistance suggested common signaling pathway between metabolic syndrome (MetS) and schizophrenia. Deficiency of kynurenine-3-monooxygenase (KMO), enzyme catalyzing formation of 3-hydroxykynurenine (3-HK) from kynurenine (Kyn), a tryptophan (Trp) metabolite, might contribute to development of MetS as suggested by non-expression of KMO genes in human fat tissue and elevated serum concentrations of Kyn and its metabolites, kynurenic (KYNA) and anthranilic (ANA) acids, in diabetic patients and Zucker fatty rats (ZFR). Markers of KMO deficiency: decreased 3-HK and elevated Kyn, KYNA and ANA, were observed in brains and spinal fluids of SP, and in brains and serum of experimental animals with genetically- or pharmacologically-induced KMO deficiency. However, elevated concentrations of ANA and decreased 3-HK were reported in serum of SP without concurrent increase of Kyn and KYNA. Present study aimed to re-assess serum Kyn metabolites (HPLC-MS) in a sub-group of SP with elevated KYNA. We found increased Kyn concentrations (by 30%) and Kyn:Trp ratio (by 20%) in serum of SP with elevated KYNA concentrations (by 40%). Obtained results and our previous data suggest that peripheral KMO deficiency might be manifested by, at least, two different patterns: elevated ANA with decreased 3-HK; and elevated KYNA and KYN. The latter pattern was previously described in type 2 diabetes patients and might underline increased predisposition of SP to development of MetS. Assessment of peripheral KMO deficiency might identify SP predisposed to MetS. Attenuation of the consequences of peripheral KMO deficiency might be a new target for prevention/treatment of obesity and diabetes in SP.

## Introduction

**Figure 1:**
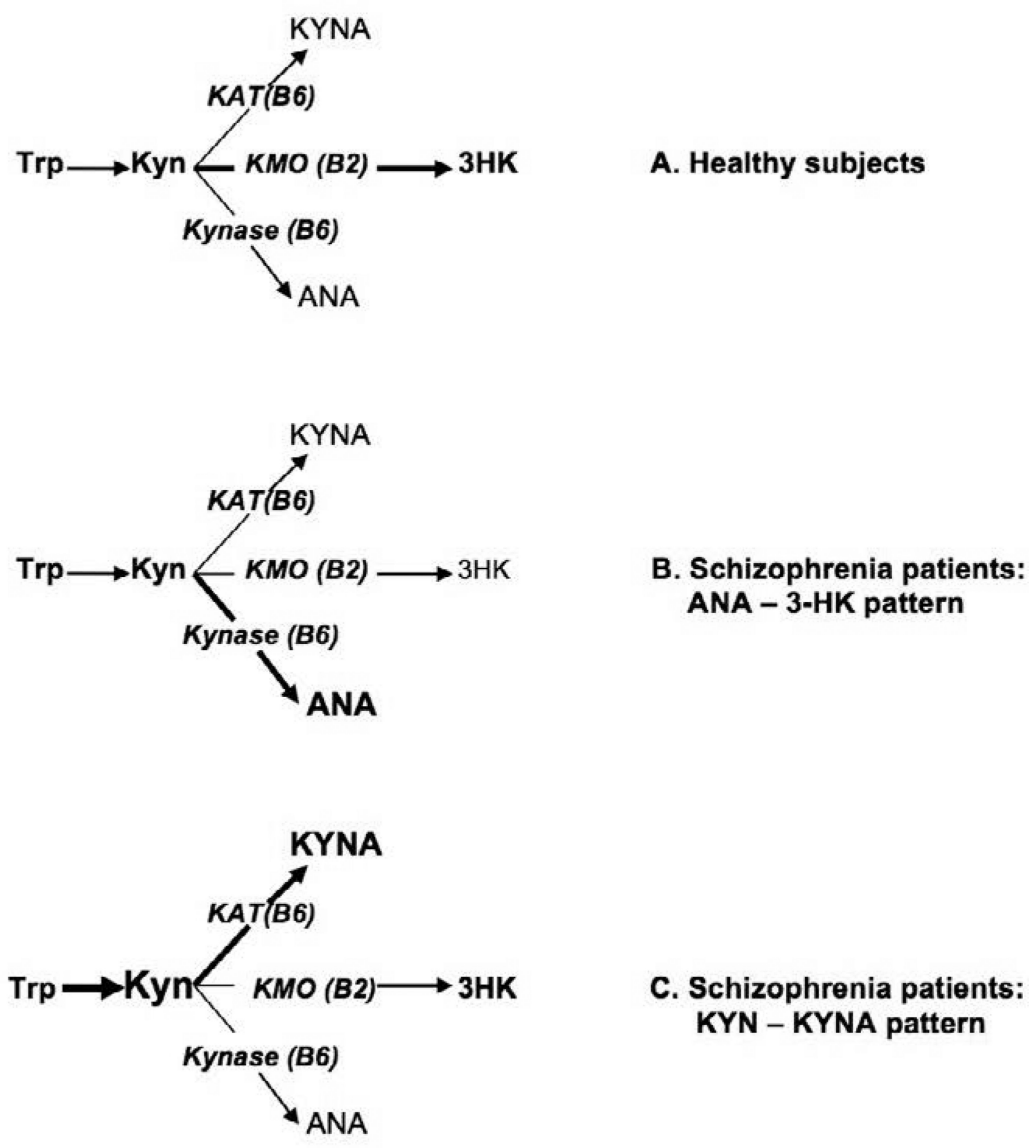
Different patterns of peripheral KMO deficiency in schizophrenia patients.

Increased predisposition of Schizophrenia Patients (SP) and their first-degree relatives to development of obesity and insulin resistance suggests common signaling pathway between schizophrenia and metabolic syndrome (MetS) [1]. Dysregulation of kynurenine (Kyn) metabolism was proposed as one of the mechanisms of MetS [2–11]. Elevated serum concentrations of Kyn and its down-stream derivative, kynurenic acid (KYNA), were observed in type 2 diabetes [12] and in Zucker Fatty Rats (ZFR), an experimrntal model of MetS [13]suggesting deficiency of kynurenine-3-monooxygenase (KMO), a key enzyme of Kyn down-stream metabolism [14]. There are converging evidences of KMO deficiency in SP. Kyn is formed from Trp during the initial phase of KP [14]. Further metabolism of KYN is trifurcated into production of 3-hydroxyKyn (3-HK), catalyzed by vitamin B2-dependent Kyn-3-monooxygenase (KMO); kynurenic acid (KYNA) and anthranilic acid (ANA), catalyzed by vitamin B6-dependent Kyn-aminotransferase (KAT) and kynureninase (Kynase), resp (Figure 1A) [15]. “KYNA hypothesis of schizophrenia” [16] was initiated by a discovery of KMO deficiency in Broadmann area of brain of schizophrenia patients (SP) [17], and was supported by findings of elevated KYNA concentrations in brains [18] and CSF [19] of SP and by observations of KYNA-induced schizophrenia-like symptoms in experimental animals [20], including disruption of pre-pulse inhibition [21] and impairment of cognitive functions [22], and damage of spinal cord myelin [23] and impairment of oligodendrocyte viability [24]. KMO deficiency increased availability of Kyn as a substrate for unsaturated enzymes, KAT and Kynase, and, therefore, shifts downstream metabolism of Kyn from formation of 3-HK toward production of KYNA and ANA [15]. It was suggested that KYNA contributed to up-regulation of brain dopamine receptors, the hall mark of schizophrenia, via its antagonism to NMDA and a7-nicotinic acetylcholine receptors [18]. Besides the brain (e.g., glial cells), Kyn, KYNA, ANA, and 3-HK are formed by peripheral tissues (e.g., macrophages, pancreatic cells, adipocytes) [5,25,26]. All four markers of KMO deficiency, i.e., elevated Kyn, KYNA and ANA and decreased 3-HK, were observed not only in brain but in serum of experimental animals with KMO deficiency, induced by vitamin B2-deficient diet [27,28] or by knockout of gene, that encodes KMO [29,30]. However, in clinical studies, only elevated ANA and decreased 3-HK concentrations were observed in serum of SP without concurrent increase of Kyn and KYNA [15,31]. Therefore, we were interested to expand our previous study [9] by assessing serum KP metabolites in a subgroup of SP with elevated KYNA.

## Materials and methods

### Patients

Overnight fasting blood samples from SP (diagnosed according to DSM-V) with serum concentrations of KYNA higher than in controls (three men and four women, age range from 38 to 56 years) were selected for analysis of Kyn and its metabolites. All patients were taking anti-psychotic medication: Abilify (three patients), Haloperidol (two patients) and Haloperidol decanoate injections (two patients).

### Healthy Subjects (Controls)

There were 12 subjects (6 females and 6 males, age range from 32 to 64 years). Study was approved by Tufts Medical Center IRB.

### Assessment of kynurenine metabolites

Serum samples were stored at −50°C until analysis. ANA, Trp, Kyn, KYNA and 3-HK concentrations were analyzed by modified HPLC–mass spectrometry method as described elsewhere [15].

### Statistical analysis

Results are presented as mean ± standard error (Trp and Kyn in µM and AA, KYNA and 3-HK in nM). Statistical significance was assessed by unpaired t test with Welch correction.

## Results

KYNA concentrations in studied SP were higher (approximately by 40%) in comparison with controls. Kyn concentrations were elevated by 30%. Kyn:Trp ratio, an indicator of activity of tryptophan-2,3-dioxygenase (TDO), the first and rate-limiting enzyme of Trp – Kyn pathway, TDO activity, was increased by 20%. There was no statistically significant difference between concentrations of Trp, 3-HK and ANA in SP and controls (Table 1).

## Discussion

Present results (together with our previously published data) suggest that peripheral KMO deficiency is a common feature of MetS and schizophrenia. Experimental data suggested, at least, four potential clinical markers of KMO deficiency: elevation of KYN, ANA, KYNA and decrease of 3-HK serum concentrations. However, only two markers, elevated ANA and decreased 3-HK serum concentrations, were reported in SP without concurrent elevation of Kyn and KYNA concentrations [15,31]. In the present study of a sub-group of SP with higher than controls KYNA concentration, we observed elevation of serum concentrations of Kyn without concurrent elevation of ANA and decrease of 3HK concentrations. Notably, we observed a significant increase of Kyn:Trp ratio, suggesting activation of Trp conversion into Kyn catalyzed either by inflammation-induced indoleamine-2,3-dioxygenase (IDO) or by stress-induced TDO [14], while successful metformin treatment of insulin resistance was reported recently to be associated with down-regulation of the Trp - Kyn pathway [32]. TDO activation was previously described in prefrontal cortex of SP [33]. Therefore, elevated serum concentration of Kyn (and Kyn:Trp ratio) might be a result of KMO deficiency and/or IDO/TDO activation. Present data and our previous report suggested the existence of, at least, two patterns of peripheral KMO deficiency in SP: elevated ANA with decreased 3-HK (without changes of Kyn and KYNA) (Figure 1B); and elevated Kyn and KYNA (without changes of ANA and 3-HK) (Figure 1C). The latter pattern of peripheral KMO deficiency, i.e., elevation of both KYN and KYNA without elevation of ANA, was described in type 2 diabetes [34] and might underline increased predisposition of SP to MetS. Notably, both astrocytes and fat tissue do not express KMO genes [35,36]. Peripherally produced Kyn, ANA and 3-HK (but not KYNA) might contribute to central pathology by crossing blood brain barrier (BBB) [37] and entering a pool of centrally formed Kyn metabolites [38]. Dysregulation of Trp – Kyn pathway was suggested as a common signaling pathway for schizophrenia and Metabolic Syndrome in schizophrenia [9,13,15]. Elevated serum concentrations of KYNA, indicative of KMO deficiency, were observed in ZFR [13], and associated with weight gain in humans [39]. KYNA and KYN are endogenous ligands to aryl hydrocarbon receptor (AHR) that regulates xenobiotic-metabolizing enzymes such as aryl hydrocarbon hydroxylase (cytochrome P450) in humans and rodents [40]. AHR over-activation promoted while AHR deficiency protected mice from diet-induced obesity [41,42]. Therefore, peripheral KMO deficiency might contribute to metabolic abnormalities in SP via activation of AHR by increased formation of down-stream Kyn metabolites. Further studies might explore the use of evaluation of serum concentrations of Kyn and its down-stream metabolites in identification of SP at risk for development of MetS. Modulation of down-stream Kyn metabolism (by, e.g., inhibitors of KYNA and ANA formation) might be a new target for prevention/treatment of obesity in SP.

## Figures and Tables

**Table 1:** Serum concentrations of Kyn metabolites in schizophrenia patients.

	Controls # (n=12)	Schizophrenia P * (n=7)	
Tryptophan (µM)	68.90 ± 2.49	74.22 ± 5.28	ns
Kynurenine (µM)	1.76 ± 0.09	2.32 ± 0.12	0.02
Kyn × 100: Trp	2.56 ± 0.35	3.47 ± 0.20	0.03
3-HK (nM)	19.55 ± 3.14	11.85 ± 4.09	ns
KYNA (nM)	35.78 ± 3.59	49.23 ± 4.02	0.02
ANA (nM)	21.65 ± 5.99	23.92 ± 5.86	ns

#) mean + standard error;

*) unpaired t test with Welch correction

Abbreviations: KYNA: kynurenic acid; ANA : anthranilic acid; 3-HK : 3-HydroxyKynurenine.

## Abbreviations

Trp: Tryptophan
Kyn: Kynurenine
KYNA: Kynurenic acid
ANA: Anthranilic Acid
3-HK: 3-HydroxyKynurenine
IDO: Indoleamine 2,3-dioxygenase
TDO: Tryptophan 2,3-dioxygenase
KMO: Kynurenine-3-Monoxygenase
KAT: Kynurenine Amino Transferase
Kynase: Kynureninase

## References

[1] Pillinger T, Beck K, Gobjila C, Donocik JG, Jauhar S (2017). Impaired Glucose Homeostasis in First-Episode Schizophrenia: A Systematic Review and Meta-analysis. JAMA Psychiatry.

[2] Brandacher G, Hoeller E, Fuchs D, Weiss HG (2007). Chronic immune activation underlies morbid obesity: is IDO a key player?. Curr Drug Metab.

[3] Oxenkrug GF (2010). Metabolic syndrome, age-associated neuroendocrine disorders, and dysregulation of tryptophan-kynurenine metabolism. Ann N Y Acad Sci.

[4] Watts SW, Shaw S, Burnett R, Dorrance AM (2011). Indoleamine 2,3-diooxygenase in periaortic fat: mechanisms of inhibition of contraction. Am J Physiol Heart Circ Physiol.

[5] Wolowczuk I, Hennart B, Leloire A, Bessede A, Soichot M (2012). Tryptophan metabolism activation by indoleamine 2,3-dioxygenase in adipose tissue of obese women: an attempt to maintain immune homeostasis and vascular tone. Am J Physiol Regul Integr Comp Physiol.

[6] Oxenkrug G (2013). Insulin resistance and dysregulation of tryptophan-kynurenine and kynurenine-nicotinamide adenine dinucleotide metabolic pathways. Mol Neurobiol.

[7] Poulain-Godefroy O, Eury E, Leloire A, Hennart B, Guillemin GJ (2013). Induction of TDO2 and IDO2 in Liver by High-Fat Feeding in Mice: Discrepancies with Human Obesity. Int J Tryptophan Res.

[8] Mangge H, Summers KL, Meinitzer A, Zelzer S, Almer G (2014). Obesity-related dysregulation of the tryptophan-kynurenine metabolism: role of age and parameters of the metabolic syndrome. Obesity (Silver Spring).

[9] Oxenkrug G (2015). 3-hydroxykynurenic acid and type 2 diabetes: implications for aging, obesity, depression, Parkinson’s disease and schizophrenia. Tryptophan Metabolism: Implications for Biological Processes, Health and Diseases, Molecular and Integrative Toxicology.

[10] Navrotskaya V, Oxenkrug G, Vorobyova L, Summergrad P (2015). Attenuation of high sucrose diet-induced insulin resistance in tryptophan 2,3-dioxygenase deficient Drosophila melanogaster vermilion mutants. Integr Obes Diabetes.

[11] Pedersen ER, Tuseth N, Eussen SJ, Ueland PM, Strand E (2015). Associations of plasma kynurenines with risk of acute myocardial infarction in patients with stable angina pectoris. Arterioscler Thromb Vasc Biol.

[12] Oxenkrug GF (2015). Increased Plasma Levels of Xanthurenic and Kynurenic Acids in Type 2 Diabetes. Mol Neurobiol.

[13] Oxenkrug G, Cornicelli J, van der Hart M, Roeser J, Summergrad P (2016). Kynurenic acid, an aryl hydrocarbon receptor ligand, is elevated in serum of Zucker fatty rats. Integr Mol Med.

[14] Schwarcz R, Bruno JP, Muchowski PJ, Wu HQ (2012). Kynurenines in the mammalian brain: when physiology meets pathology. Nat Rev Neurosci.

[15] Oxenkrug G, van der Hart M, Roeser J, Summergrad P (2016). Anthranilic Acid: A Potential Biomarker and Treatment Target for Schizophrenia. Ann Psychiatry Ment Health.

[16] Erhardt S, Schwieler L, Nilsson L, Linderholm K, Engberg G (2007). The kynurenic acid hypothesis of schizophrenia. Physiol Behav.

[17] Wonodi I, Stine CO, Sathyasaikumar KV, Robert RC, Mitchell BD (2011). Downregulated Kynurenine 3-Monooxygenase Gene Expression and Enzyme Activity in Schizophrenia and Genetic Association With Schizophrenia Endophenotypes. Arch Gen Psychiatry.

[18] Schwarcz R, Rassoulpour A, Wu HQ, Medoff D, Tamminga CA (2001). Increased cortical kynurenate content in schizophrenia. Biol Psychiatry.

[19] Erhardt S, Blennow K, Nordin C, Skogh E, Lindström LH (2001). Kynurenic acid levels are elevated in the cerebrospinal fluid of patients with schizophrenia. Neurosci Lett.

[20] Erhardt S, Pocivavsek A, Repici M, Liu XC, Imbeault S (2016). Adaptive and Behavioral Changes in Kynurenine 3-Monooxygenase Knockout Mice: Relevance to Psychotic Disorders. Biol Psychiatry.

[21] Erhardt S, Schwieler L, Emanuelsson C, Geyer M (2004). Endogenous kynurenic acid disrupts prepulse inhibition. Biol Psychiatry.

[22] Chess AC, Simoni MK, Alling TE, Bucci DJ (2007). Elevations of endogenous kynurenic acid produce spatial working memory deficits. Schizophr Bull.

[23] Dabrowski W, Kwiecien JM, Rola R, Klapec M, Stanisz GJ (2015). Prolonged subdural infusion of kynurenic acid is associated with dose-dependent myelin damage in the rat spinal cord. PLoS One.

[24] Langner E, Lemieszek MK, Kwiecień JM, Rajtar G, Rzeski W (2016). Kynurenic Acid Induces Impairment of Oligodendrocyte Viability: On the Role of Glutamatergic Mechanisms. Neurochem Res.

[25] Liu JJ, Raynal S, Bailbé D, Gausseres B, Carbonne C (2015). Expression of the kynurenine pathway enzymes in the pancreatic islet cells. Activation by cytokines and glucolipotoxicity. Biochim Biophys Acta.

[26] Paluszkiewicz P, Zgrajka W, Saran T, Schabowski J, Piedra JL (2009). High concentration of kynurenic acid in bile and pancreatic juice. Amino Acids.

[27] Verjee ZH (1975). Tryptophan metabolism in baboons: effect of riboflavin and pyridoxine deficiency. Acta Vitaminol Enzymol.

[28] Charconnet-Harding F, Dalgliesh CE, Neuberger A (1953). The relation between riboflavin and tryptophan metabolism, studied in the rat. Biochem J.

[29] Giorgini F, Huang SY, Sathyasaikumar KV, Notarangelo FM, Thomas MA (2013). Targeted deletion of kynurenine 3-monooxygenase in mice: a new tool for studying kynurenine pathway metabolism in periphery and brain. J Biol Chem.

[30] Tashiro T, Murakami Y, Mouri A, Imamura Y, Nabeshima T (2015). Kynurenine 3-monooxygenase is implicated in antidepressants-responsive depressive-like behaviors and monoaminergic dysfunctions. Behav Brain Res.

[31] Fazio F, Lionetto L, Curto M, Iacovelli L, Cavallari M (2015). Xanthurenic Acid Activates mGlu2/3 Metabotropic Glutamate Receptors and is a Potential Trait Marker for Schizophrenia. Sci Rep.

[32] Muzik O, Burghardt P, Yi Z, Kumar A, Seyoum B (2017). Successful metformin treatment of insulin resistance is associated with down-regulation of the kynurenine pathway. Biochem Biophys Res Commun.

[33] Miller CL, Llenos IC, Cwik M, Walkup J, Weis S (2008). Alterations in kynurenine precursor and product levels in schizophrenia and bipolar disorder. Neurochem Int.

[34] Korstanje R, Deutsch K, Bolanos-Palmieri P, Hanke N (2016). Loss of Kynurenine 3-Mono-oxygenase Causes Proteinuria. J Am Soc Nephrol.

[35] Guillemin GJ, Smith DG, Kerr SJ, Smythe GA, Kapoor V (2000). Characterisation of kynurenine pathway metabolism in human astrocytes and implications in neuropathogenesis. Redox Rep.

[36] Favennec M, Hennart B, Caiazzo R, Leloire A, Yengo L (2015). The Kynurenine Pathway is Activated in Human Obesity and Shifted Toward Kynurenine Monooxygenase Activation. Obesity.

[37] Fukui S, Schwarcz R, Rapoport SI, Takada Y, Smith QR (1991). Blood-brain barrier transport of kynurenines: implications for brain synthesis and metabolism. J Neurochem.

[38] Baran H, Schwarcz R (1990). Presence of 3-hydroxyanthranilic acid in rat tissues and evidence for its production from anthranilic acid in the brain. J Neurochem.

[39] Zhao J, Shen D, Djukovic C, Daniel-MacDougall H, Gu X (2016). Metabolomics-identified metabolites associated with body mass index and prospective weight gain among Mexican American women. Obes Sci Pract.

[40] DiNatale BC, Murray IA, Schroeder JC, Flaveny CA, Lahoti TS (2010). Kynurenic acid is a potent endogenous aryl hydrocarbon receptor ligand that synergistically induces interleukin-6 in the presence of inflammatory signaling. Toxicol Sci.

[41] Xu CX, Wang C, Zhang ZM, Jaeger CD, Krager SL (2015). Aryl hydrocarbon receptor deficiency protects mice from diet-induced adiposity and metabolic disorders through increased energy expenditure. Int J Obes (Lond).

[42] Moyer BJ, Rojas IY, Kerley-Hamilton JS, Hazlett HF, Nemani KV (2016). Inhibition of the aryl hydrocarbon receptor prevents Western diet-induced obesity. Model for AHR activation by kynurenine via oxidized-LDL, TLR2/4, TGFβ, and IDO1. Toxicol Appl Pharmacol.

